# *Bacillus amyloliquefaciens* attenuates the intestinal permeability, oxidative stress and endoplasmic reticulum stress: transcriptome and microbiome analyses in weaned piglets

**DOI:** 10.3389/fmicb.2024.1362487

**Published:** 2024-05-13

**Authors:** Junmeng Yuan, Hongling Meng, Yu Liu, Li Wang, Qizhen Zhu, Zhengyu Wang, Huawei Liu, Kai Zhang, Jinshan Zhao, Weifen Li, Yang Wang

**Affiliations:** ^1^College of Animal Science and Technology, Qingdao Agricultural University, Qingdao, China; ^2^College of Animal Sciences, Zhejiang University, Hangzhou, China

**Keywords:** *Bacillus amyloliquefaciens*, intestinal health, endoplasmic reticulum stress, oxidative stress, transcriptome, microbiome

## Abstract

Endoplasmic reticulum (ER) stress is related to oxidative stress (OS) and leads to intestinal injury. *Bacillus amyloliquefaciens* SC06 (SC06) can regulate OS, but its roles in intestinal ER stress remains unclear. Using a 2 × 2 factorial design, 32 weaned piglets were treated by two SC06 levels (0 or 1 × 10^8^ CFU/g), either with or without diquat (DQ) injection. We found that SC06 increased growth performance, decreased ileal permeability, OS and ER stress in DQ-treated piglets. Transcriptome showed that differentially expressed genes (DEGs) induced by DQ were enriched in NF-κB signaling pathway. DEGs between DQ- and SC06 + DQ-treated piglets were enriched in glutathione metabolism pathway. Ileal microbiome revealed that the SC06 + DQ treatment decreased *Clostridium* and increased *Actinobacillus*. Correlations were found between microbiota and ER stress genes. In conclusion, dietary SC06 supplementation increased the performance, decreased the permeability, OS and ER stress in weaned piglets by regulating ileal genes and microbiota.

## 1 Introduction

During weaning, piglets encounter various stressors, such as the sudden separation from their mothers, mixing with other litters, and eating the less digestible feed (Lallès et al., 2007). These stressors can generate excessive amounts levels of reactive oxygen species (ROS) and have the potential to cause oxidative stress (OS) (Zheng et al., 2017). The small intestine performs many physiological functions, including digestion, absorption and immunoregulation (Pluske et al., 1997). In the ileal lumen, alkaline intestinal fluid that containing a variety of enzymes can break down digesta into glucose, amino acids, and fatty acids (Malik et al., 2023). Nutrients, such as bile salts and vitamin B_12_ can be absorbed by ileum (Hyder et al., 2023). In addition, immune cells in ileum can also participate in the immune response (Chen et al., 2021). It is reported that OS leads to histological and biochemical damages of small intestine, leading to post-weaning diarrhea and growth retardation in weaned piglets (Su et al., 2023).

OS and ROS production are thought to be components of endoplasmic reticulum (ER) stress. The ER is the largest organelle in the cell and is a major site for protein metabolism, lipid synthesis, carbohydrate metabolism and calcium storage (Reid and Nicchitta, 2015; Westrate et al., 2015). Report also indicated that mitigation of ER stress may be an effective strategy to alleviate OS and growth retardation (Jing et al., 2023). The ER unfolded protein response (ER-UPR) appears to be the representative biomarker of ER stress. Accumulation and aggregation of unfolded proteins can impair normal cellular function and result in cell death (Wang et al., 2019). Studies showed that various stimuli, such as porcine epidemic diarrhea virus, *Escherichia coli* and LPS, could aggravate the ER stress in weaned piglets (Jiang et al., 2017; Chen et al., 2022; Liu et al., 2022).

Strain-specific probiotics have been reported to elevate the growth performance (Wang et al., 2017a), antioxidant capacity (Wang et al., 2012) and intestinal health (Hu et al., 2018) of weaned piglets. Moreover, recent study has also demonstrated that probiotics could ameliorate the ER stress. Yang et al. (2020) reported that ER stress in the ileum of *Salmonella*-infected piglets was significantly alleviated after intragastric injection of *Lactobacillus johnsonii* L531. Zhang et al. (2021) suggested that *Lactobacillus johnsonii* attenuated *Citrobacter rodentium*-induced colitis by regulating ER stress in mice. Chen et al. (2024) showed that dietary supplementation with *Bacillus subtilis* HW2 significantly alleviated ER stress in the ileum of broilers with necrotizing enteritis. Our previous studies have proved that *Bacillus amyloliquefaciens* SC06 (SC06) improved the antioxidant capacity and intestinal barrier function of piglets and IPEC-1 cells (Wang et al., 2017b; Du et al., 2018). However, whether SC06 can attenuate the intestinal ER stress level in weaned piglets remains unclear.

Gut microbiota can affect the intestinal redox state by producing metabolites such as SCFAs and polyphenolic metabolites (Zhao et al., 2023). Lee et al. (2016) showed that the changes in gut microbiota are closely related to OS in weaned piglets. Fu et al. (2021) suggested that the relative abundance of Firmicutes and Actinobacteria, the genus *Ruminococcaceae UCG-005*, and members of *Eubacterium coprostanoligenes* was increased in the intestinal tract of oxidatively-stressed piglets.

Hence, the present study aimed to reveal the effects of dietary SC06 supplementation on the growth performance, ileal barrier function, OS and ER stress in diquat (DQ)-treated weaned piglets by using ileal mucosal transcriptome and microbiome analyses.

## 2 Materials and methods

### 2.1 Experimental design

A total of 32 weaned male piglets (Duroc × Landrace × Yorkshire, 29-day-old) with similar initial body weights (7.51 ± 0.39 kg) were assigned to a 2 × 2 factorial design with two SC06 levels (0 or 1 × 10^8^ CFU/g) and two DQ levels (0 or 10 mg/kg body weight). Therefore, the 4 treatments were: control group (Con), basal diet; DQ group, basal diet and intraperitoneal injection of 10 mg/kg DQ; SC06 group, basal diet containing 1 × 10^8^ CFU/g SC06; SC06 + DQ group, basal diet containing 1 × 10^8^ CFU/g SC06 and intraperitoneal injection of DQ (10 mg/kg body weight), with 8 piglets per pen. The dosage of DQ was based on Zheng et al. (2017). At day 22 of experiment, DQ (diquat dibromide monohydrate, PS365; Sigma, United States) was intraperitoneally injected. The SC06 powder was pre-mixed with 1 kg of the basal diet and successively mixed into the remaining diet. The animal study was reviewed and approved by the Animal Care and Use Committee of Qingdao Agricultural University (protocol number 20221125374). We have followed the ARRIVE guidelines for reporting animal research. The basal diet of piglets was prepared according to NRC 2012 and the composition and nutrient levels were listed in Table 1.

**Table 1:** Composition and nutrient levels of basal diets (air-dry basis) %.

Item	Content
**Ingredients**	
Corn	60.00
Soybean meal	12.30
Extruded soybean	3.70
Fish meal	3.46
Whey powder	5.00
Soybean protein concentrate	6.60
Soybean oil	2.60
Sucrose	3.00
Limestone	0.60
CaHPO_4_	0.90
NaCl	0.10
*L*-Lys·HCl	0.50
*DL*-Met	0.10
*L*-Thr	0.10
*L*-Trp	0.04
Chloride choline	0.10
Premix^a^	0.50
Acidifier	0.40
Total	100.00
**Nutrient levels** ^b^	
DE/(MJ/kg)	14.73
CP (%)	19.03
Ca (%)	0.79
AP (%)	0.37
D-Lys (%)	1.33
D-Met (%)	0.38
Thr (%)	0.74
Trp (%)	0.23

a The premix provided the following per kg of diets: VA 2000 IU, VD_3_ 300 IU, VE 20 IU, VB_1_ 2.0 mg, VB_2_ 4.0 mg, VB_6_ 5.0 mg, VB_12_ 0.02 mg, VK_3_ 3.0 mg, biotin 0.1 mg, folic acid 0.5 mg, pantothenic acid 15.0 mg, nicotinic acid 0.75 mg, Cu (as copper sulfate) 25 mg, Fe (as ferrous sulfate) 100 mg, Mn (as manganese sulfate) 10 mg, Zn (as zinc sulfate) 100 mg, I (as potassium iodide) 0.30 mg, Se (Na_2_SeO_3_) (as sodium selenite) 100 mg, phytase, 100 IU.

b The nutrient levels were calculated values.

### 2.2 Sample collection

At the 29th day of the trial, blood samples were collected from the vena cava anterior and were centrifuged for 10 min at 4°C (3,000 × g, Centrifuge 5804R, Eppendorf, Germany) to obtain the serum. Serum samples were stored at −20°C. Mid-ileal segments were carefully dissected and rinsed with sterilized saline. Part of the ileal segment was cut and fixed in 4% paraformaldehyde solution. Then, cotton swabs were used to wipe the ileal mucosa in order to obtain the mucosal bacteria. The swab tips were collected in 1.5 mL centrifuge tubes. Moreover, ileal mucosa samples were also gently scraped off. The swab tips and ileal mucosa samples were placed in liquid nitrogen immediately and then stored at −80°C for further analysis.

### 2.3 Biochemical analyses

Ileal mucosa samples were homogenized with ice-cold physiologic saline (1:10, w/v) and centrifuged at 2,000 × g for 10 min (4°C), and the protein concentration in the supernatant was determined using a BCA Protein Concentration Determination Kit (Beyotime Biotechnology Co., Ltd., Shanghai, China). The concentrations of malondialdehyde (MDA, Cat. YJ54526), D-Lactate (Cat. YJ75986) and the diamine oxidase (DAO, Cat. YJ98519) and the activities of catalase (CAT, Cat. YJ22695), superoxide dismutase (SOD, Cat. YJ45986) and glutathione peroxidase (GSH-Px, Cat. YJ36985) were determined using by Enzyme-linked immunosorbent assay (ELISA) kits (Enzyme-Linked Biotechnology Co., Ltd., Shanghai, China). The level of ROS was determined by ELISA kit (ROS, Cat. MM-121201) from Meimian Industrial Co., Ltd. (Jiangsu, China). The experimental procedures were performed in strict accordance with the manufacturer’s instructions. The absorbance was read by a microplate reader (SpectraMax iD3, Molecular Devices, Shanghai, China).

### 2.4 Hematoxylin–eosin staining

The ileal segments were embedded in paraffin, and the section of each sample was mounted on glass slides for hematoxylin–eosin (HE) staining. The villus was observed under an OLYMPUS microscope (OLYMPUS, Japan) using the HMIAS-2000 (HMIAS, China) image analysis system. Villus height and crypt depth were measured according to the method of Shan et al. (2019).

### 2.5 Transmission electron microscope

The ileal tissue was cut (1 mm^3^) and fixed with electron microscope fixative (Xavier Biotechnology Co., Ltd., Wuhan, China). The ultrathin sections were cut using a ultramicrotome (Leica EM UC7, Wetzlar, Germany) and stained with uranyl acetate. Electron micrographs of the samples were then captured by the transmission electron microscope (TEM) (HITACHI HT7700 120 kV, Tokyo, Japan).

### 2.6 Real-time quantitative PCR

RNA extraction and real-time quantitative PCR (RT-qPCR) were performed according to our previous study (Yuan et al., 2023a). In brief, total RNA was extracted from ileal mucosa using Trizol reagent (Tiangen Biochemical Technology Co., Ltd., Beijing, China). RT-qPCR was then performed by using the TB Green^®^ Premix Ex Taq^™^ kit (TaKaRa) and BioRad CFX96^™^ Real-Time PCR system (Bio-Rad Laboratories, Hercules, CA). The thermal cycling protocol and primer design methods were referred to our previous report (Yuan et al., 2023b). The forward and reverse primers of the genes are presented in Supplementary Table S1. Glyceraldehyde-3-phosphate dehydrogenase (*GAPDH*) and *β-actin* as housekeeping genes were used to normalize target genes transcript levels.

### 2.7 Western blotting

The protocol was based on previous study (Liu et al., 2019). In brief, total protein was extracted from ileal mucosa and the protein concentration was measured by BCA kit (Beyotime Biotechnology Co., Ltd., Shanghai, China). Protein samples were separated by electrophoresis on SDS-PAGE and transferred electrophoretically to a nitrocellulose membranes membrane. The membrane was blocked and then incubated with primary antibodies overnight (4°C). The membrane was rinsed and then incubated with the secondary antibodies for 60 min at room temperature. The antibodies, including *β*-actin (1:1000, Cat. GB11001), GRP78 (1:500, Cat. GB11098), PERK (1:500, Cat. GB11510), ATF4 (1:500, Cat. GB11157), Claudin-1 (1:500, Cat. GB112543), Occludin (1:1000, Cat. GB11149) and ZO-1 (1:500, Cat. GB111402) were purchased from Servicebio Technology Co., Ltd. (Wuhan, China) and photoshop-PERK (p-PERK) was obtained from Affinity Biosciences Co., Ltd. (OH, United States). The blots were then developed with an ECL detection system (Invitrogen iBright FL1000, Thermo Fisher Scientific, New York) as per the manufacturer’s instructions. Image J software (National Institutes of Health, Bethesda, United States) was used to quantitate relative protein expression.

### 2.8 Total RNA extraction, mRNA library construction and sequencing

Total RNA from ileal mucosa was purified by ethanol precipitation and CTAB-PBIOZOL reagent according to the manufacturer’s instructions. Mucosal samples were ground with liquid nitrogen. The ground powder sample was transferred to 1.5 mL of preheated 65°C CTAB-pBIOZOL reagent, and then the sample was incubated in a Thermo mixer for 15 min at 65 ° C to allow complete dissociation of the nucleoprotein complex. After centrifuge at 12,000 × g for 5 min at 4°C, 400 μL of chloroform was added in the supernatant and centrifuged at 12,000 × g for 10 min at 4°C. The supernatant was transferred to a new 2 mL tube. Then, 700 μL acidic phenol and 200 μL chloroform were added followed by centrifuging 12,000 × g for 10 min at 4°C. Equal volume of aqueous phase of chloroform was added in aqueous phase and centrifuged at 12,000 × g for 10 min at 4°C. The supernatant was collected and equal volume of supernatant of isopropyl alcohol was added and placed at −20°C for 2 h for precipitation. After that, the mix was centrifuged at 12,000 × g for 20 min at 4°C and then supernatant was removed. After washing with 1 mL of 75% ethanol, the RNA pellet was air-dried in the biosafety cabinet and was dissolved by adding 50 μL of DEPC-treated water. Subsequently, total RNA was identified and quantified using the Nano Drop and Agilent 2100 bioanalyzer (Thermo Fisher Scientific, MA, United States).

mRNA was purified using oligo(dT) magnetic beads. Purified mRNA was cleaved into small fragments with fragmentation buffer at the appropriate temperature. Then first-strand cDNA was generated using random hexamer-primed reverse transcription, followed by a second-strand cDNA synthesis. Afterwards, A-Tailing Mix and RNA Index Adapters were added by incubating to end repair. The cDNA fragments obtained from previous step were amplified by PCR, and products were purified by Ampure XP Beads, then dissolved in EB solution. The product was validated for quality control on an Agilent 2100 bioanalyzer. The double-stranded PCR products from the previous step were denatured by heat with the splinted oligonucleotide sequence and cycled to obtain the final library. Single strand circle DNA (ssCir DNA) was formatted into the final library.

Qualified libraries were denatured to single-stranded PCR products. The reaction system and cycle program were subsequently configured and set up. Single-stranded cyclized products were produced, whereas non-cyclized linear DNA molecules were digested. Single-stranded circular DNA molecules were replicated by rolling cycle amplification technology to generate DNA nanospheres (DNBS) containing multiple copies of DNA. Then, DNB of sufficient quality was loaded into patterned nanoarrays using high-intensity DNA nanochip technology and sequenced by combinatorial probe anchored synthesis (cPAS).

### 2.9 Sequencing data analysis

Fastp was used to filter raw data, mainly to remove reads with adapters. When the N content of any sequencing reads exceeded 10% of the base number of the reads, the paired reads were removed. Paired bases were removed when the number of low-quality bases (*Q* ≤ 20) exceeded 50% of the total number of bases. All subsequent analyses were based on clean reads.

The reference genome and its annotation files were downloaded from the website.^1^ Feature Counts was used to calculate the gene alignments and then calculated the fragments per kilobase of exon model per million mapped fragments of each gene based on the gene length. DESeq2 was used to analyze the differential expression between the two groups, and the *p*-value was corrected using the Hochberg method. The corrected *p*-value <0.05 and |log_2_foldchange| ≥1 were used as a threshold expressed significant difference. Enrichment analysis was performed based on hypergeometric test. KEGG was tested for hypergeometric distribution by pathway. All genes raw data has been deposited in the NCBI with the Bioproject ID PRJNA1021385.

### 2.10 DNA extraction and microbiological analysis

The fecal genomic DNA extraction kit of TIANamp stool DNA kit (Tiangen Biotech Co., Ltd., Beijing, China) was used to extract the DNA of mucosa bacteria, and the experiment was carried out according to the instructions. DNA was checked for integrity and quantified. The V3–V4 region was amplified by PCR using 338F (5′-ACTCCTACGGGAGGCAGCA-3′) and 806R (5′-GGACTACHVGGGTWTCTAAT-3′) primers and finally sent to Illumina MiSeq platform for sequencing. The original tags were quality filtered using QIIME2 2019.4 software and integrated through FLASH software. After removing the chimeric sequences, the ASVs (amplicon sequence variants) were clustered by 97% similarity using uCLUST 1.2.22, and the representative sequences of each cluster were annotated according to the SILVA database. Indices of alpha diversity (Chao1, Simpson, Shannon, Pieiou_e, Observed_species, Faith_pd, Goods_coverage) of the samples were evaluated. Principal coordinate analysis (PCoA) was undertaken for all samples following analysis through application of Bray–Curtis dissimilarity and unweighted UniFrac using R packages (3.2.0).

Microbial functions were predicted by PICRUSt2 (Phylogenetic investigation of communities by reconstruction of unobserved states) KEGG^2^ databases. All DNA raw data was submitted to the NCBI database under BioProject ID PRJNA1012569.

### 2.11 Statistical analysis

The effects of SC06, DQ and their interactions on biochemical parameters were assessed using analysis of variance (ANOVA) and the general linear model procedure using IBM SPSS Statistics 18.0 (IBM Corp., Armonk, NY, United States). Multiple mean comparisons were performed using univariate ANOVA and Duncan’s multiple range test. All data was graphed by GraphPad Prism 8 (GraphPad Software, La Jolla, CA) and was expressed as the mean and standard error of the mean (SEM). The online analysis platform Biodeep^3^ was used to conduct Pearson’s correlation analysis between ER stress-related genes and bacterial genus abundance. All means and comparisons were considered statistically significant at *p* < 0.05.

## 3 Results

### 3.1 SC06 supplementation improved the growth performance of weaned piglets

The DQ injection significantly decreased the ADG (*p* < 0.01). The SC06 supplementation increased the ADG (*p* = 0.03). Moreover, the interaction between DQ and SC06 (SC06 × DQ) reversed DQ-induced changes in ADG (*p* = 0.04) (Table 2).

**Table 2:** Effect of SC06 on growth performance in weaned piglets.

Item	Initial weight (kg)	Final weight (kg)	ADG (g)
Con	7.49	21.53	547.03^a^
DQ	7.49	19.16	405.36^b^
SC06	7.52	22.21	551.79^a^
SC06 + DQ	7.52	21.81	508.93^a^
SEM	0.39	1.22	39.65
Main effect			
SC06			
−			476.19
+			530.36
DQ			
−			549.41
+			457.14
*p*-value			
SC06			0.03
DQ			<0.01
Interaction			0.04

Mean values within a row with unlike letters are significantly different (*p* < 0.05). ADG, average daily gain; Con, control diet; DQ, control diet plus diquat injection; SC06, control diet containing 1 × 10^8^ CFU/g *Bacillus amyloliquefaciens* SC06; SC06 + DQ, control diet containing 1 × 10^8^ CFU/g *Bacillus amyloliquefaciens* SC06 plus diquat injection, *n* = 6.

### 3.2 SC06 supplementation improved the ileal morphology and decreased the ileal permeability of weaned piglets

**Figure 1:**
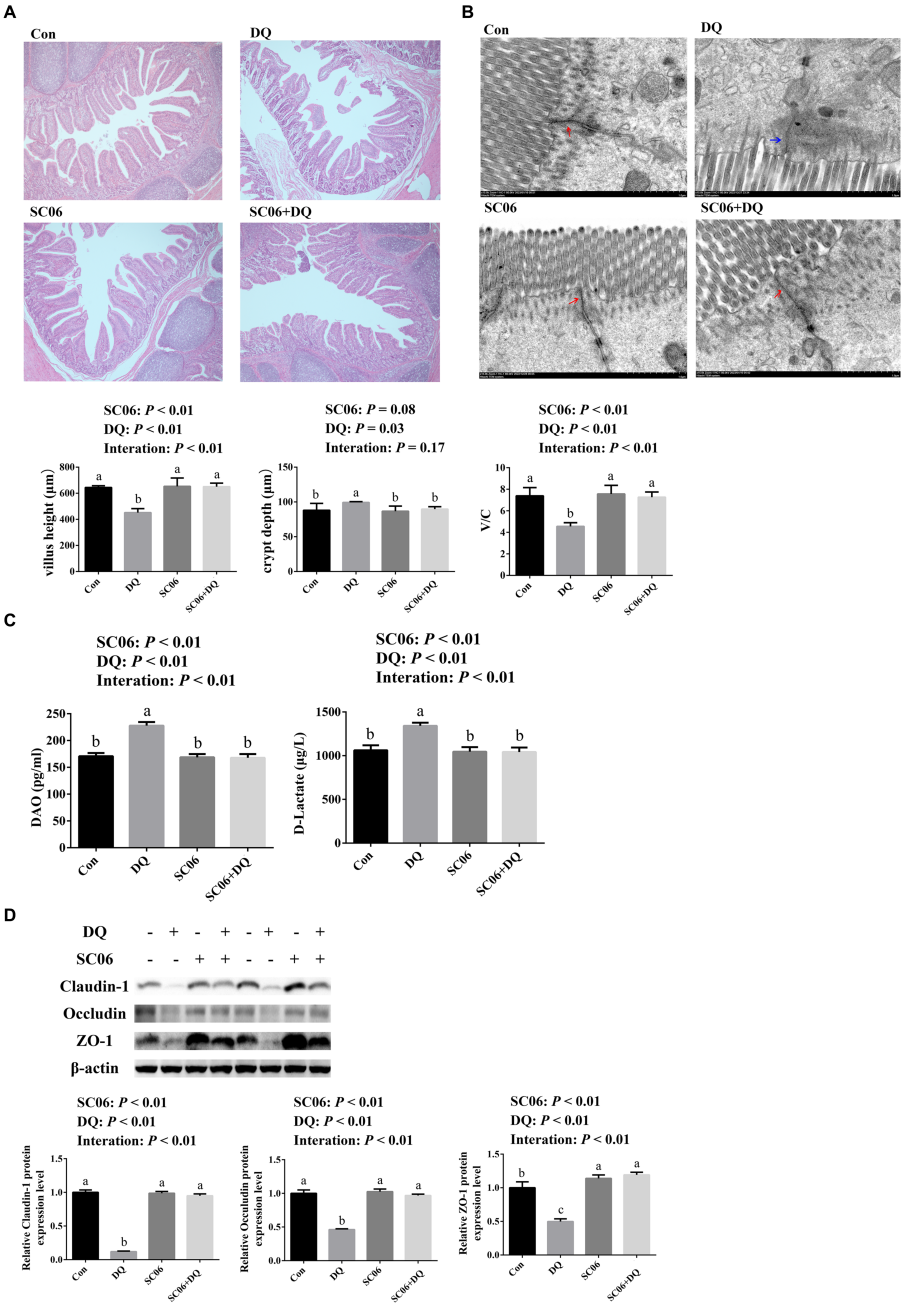
Effects of SC06 on the ileal morphology and permeability of piglets. **(A)** HE staining sections, images were photographed under 50× magnification, *n* = 6. **(B)** TEM section, images were photographed under 15 k× magnification, *n* = 3. **(C)** Serum DAO and D-lactate levels, *n* = 6. **(D)** Expressions of tight junction proteins, *n* = 3. ^a,b,c^Mean value within a row with no common superscript differ significantly (*p* < 0.05). Red arrows indicate normal tight junctions and blue arrows indicate abnormal tight junctions, V/C, villus height/crypt depth; DAO, diamine oxidase; ZO-1, zona occludens 1; Con, control diet; DQ, control diet plus diquat injection; SC06, control diet containing 1 × 10^8^ CFU/g *Bacillus amyloliquefaciens* SC06; SC06 + DQ, control diet containing 1 × 10^8^ CFU/g *Bacillus amyloliquefaciens* SC06 plus diquat injection.

The ileal morphology was explored by HE staining and TEM. HE staining results showed that DQ injection significantly decreased the villus height and V/C value (*p* < 0.01) and increased the crypt depth (*p* = 0.03). The SC06 supplementation increased villus height and V/C value (*p* < 0.01). Moreover, SC06 × DQ increased the villus height and V/C value (*p* < 0.01) in DQ-injected piglets (Figure 1A). According to the TEM analysis, we found that the tight junctions were clear and complete in the Con, SC06 and SC06 + DQ groups, but the tight junctions were not clear and the paracellular spaces were expanded in the DQ group (Figure 1B). Furthermore, the serum DAO and D-lactate levels were elevated by the DQ injection (*p* < 0.01). The SC06 supplementation decreased the levels of DAO and D-lactate (*p* < 0.01). SC06 × DQ also lowered the levels of DAO and D-lactate in DQ-treated piglets (Figure 1C). Protein expressions of tight junctions showed that the DQ treatment significantly down-regulated the expressions of claudin-1, occludin and ZO-1 (*p* < 0.01). The SC06 supplementation up-regulated the expressions of claudin-1, occludin and ZO-1 (*p* < 0.01). Moreover, SC06 × DQ increased these protein expressions in DQ-treated piglets (Figure 1D).

### 3.3 SC06 supplementation attenuated the ileal OS of weaned piglets

**Figure 2:**
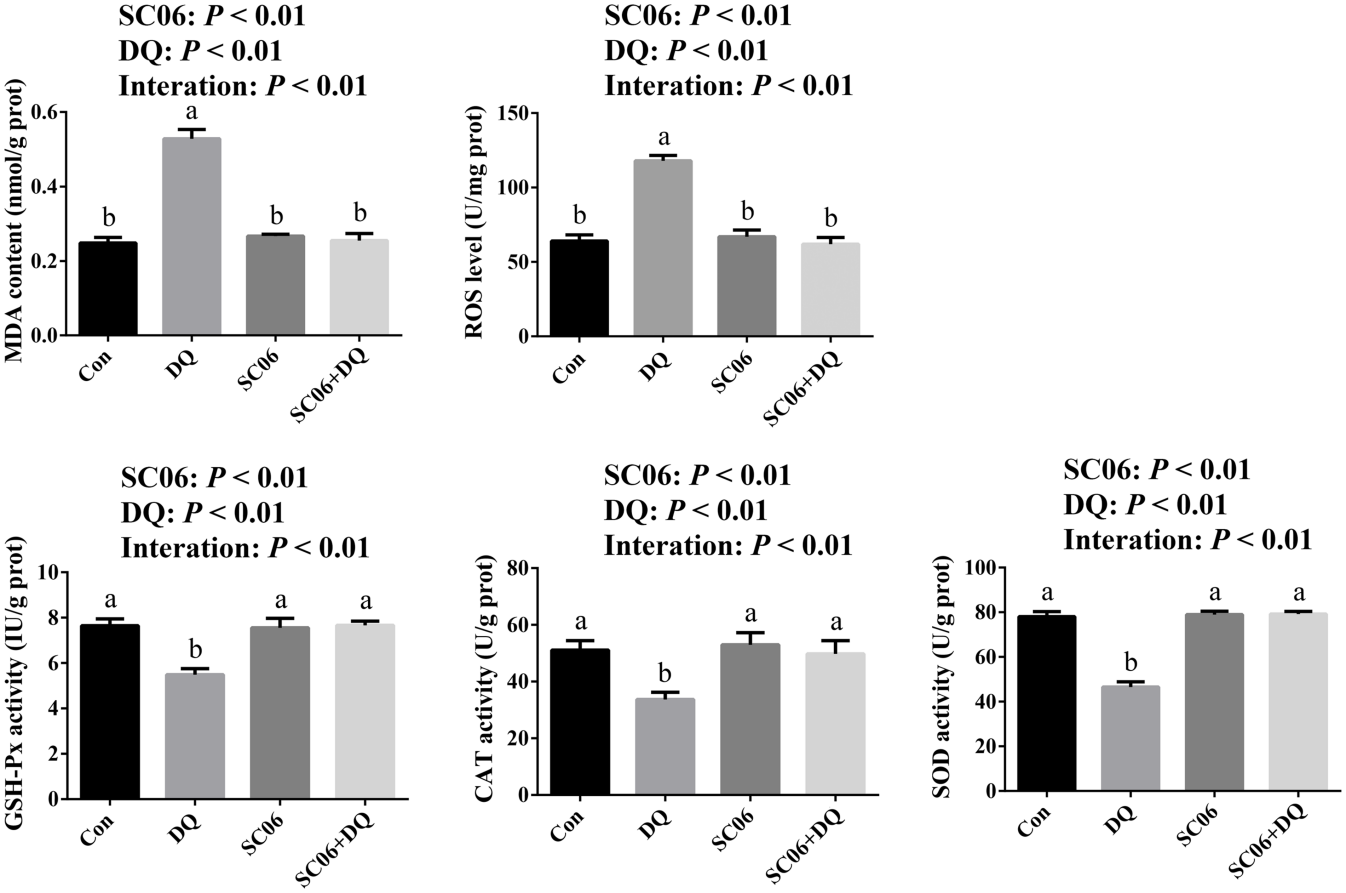
Effects of SC06 on the ileal oxidative stress of piglets. ^a,b^Mean value within a row with no common superscript differ significantly (*p* < 0.05), *n* = 6. ROS, reactive oxygen species; MDA, malondialdehyde; GSH-Px, glutathione peroxidase; CAT, catalase; SOD, superoxide dismutase; Con, control diet; DQ, control diet plus diquat injection; SC06, control diet containing 1 × 10^8^ CFU/g *Bacillus amyloliquefaciens* SC06; SC06 + DQ, control diet containing 1 × 10^8^ CFU/g *Bacillus amyloliquefaciens* SC06 plus diquat injection.

The DQ injection elevated the levels of MDA and ROS (*p* < 0.01), and decreased activities of CAT, SOD and GSH-Px (*p* < 0.01). The SC06 supplementation decreased the levels of MDA and ROS (*p* < 0.01), and increased activities of CAT, SOD and GSH-Px (*p* < 0.01). Moreover, SC06 × DQ reversed the changes in MDA, ROS, CAT, SOD and GSH-Px in DQ-treated piglets (Figure 2).

### 3.4 SC06 supplementation attenuated the ileal ER stress of weaned piglets

**Figure 3:**
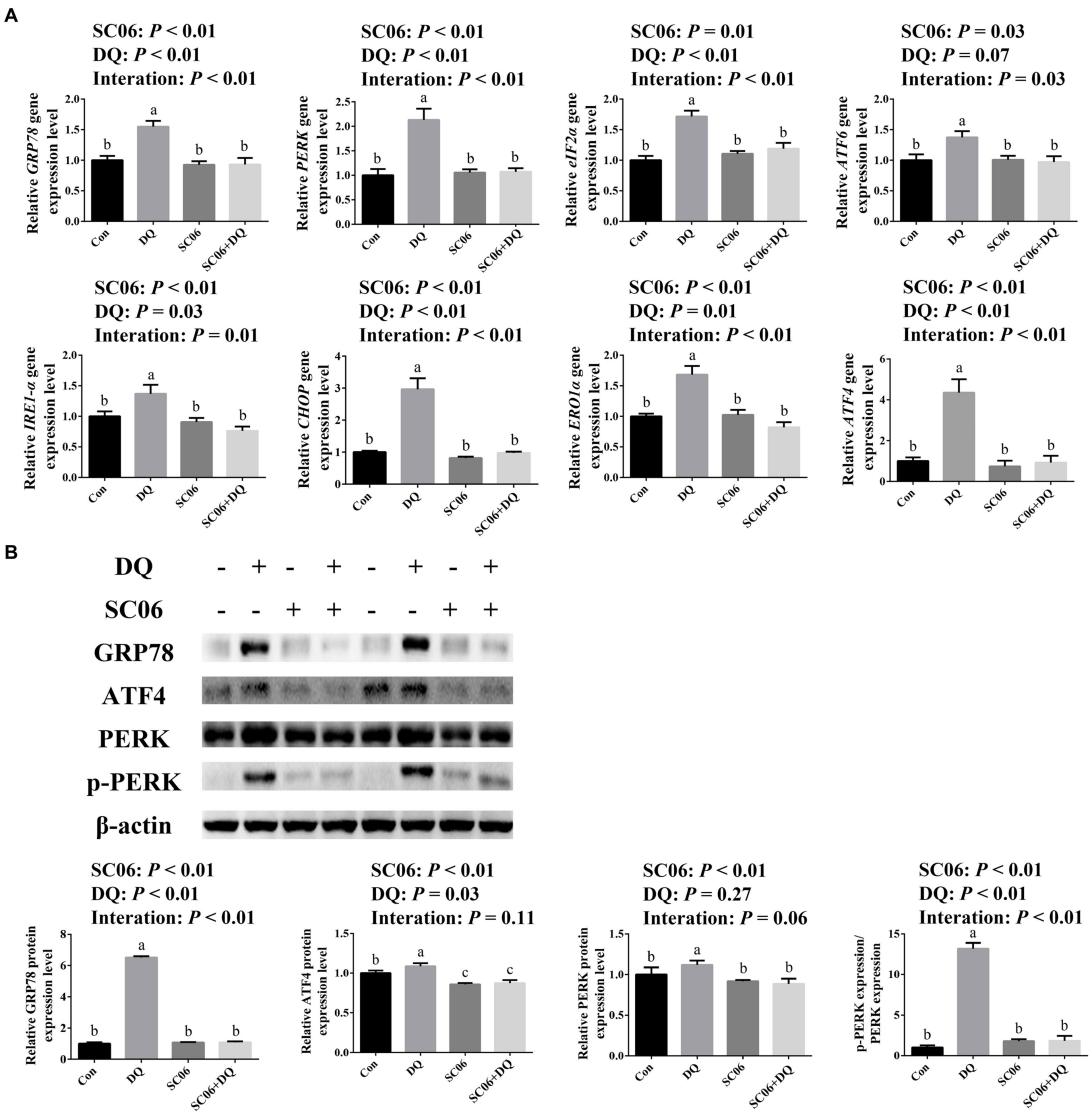
Effects of SC06 on the ileal ER stress of piglets. **(A)** Expressions of ER stress-related genes, *n* = 6. **(B)** Expressions of ER stress-related proteins, *n* = 3. ^a,b,c^Mean value within a row with no common superscript differ significantly (*p* < 0.05). *GRP78*, glucose-regulated protein 78; *PERK*, protein kinase RNA-like endoplasmic reticulum kinase; *eIF2α*, phosphorylation of eukaryotic initiation factor-2*α*; *ATF6*, activating transcription factor 6; *IRE1-α*, inositol-requiring enzyme 1*α*; *CHOP*, C/EBP homologous protein; *ERO1α*, endoplasmic reticulum oxidoreductin 1*α*; *ATF4*, activating transcription factor 4; Con, control diet; DQ, control diet plus diquat injection; SC06, control diet containing 1 × 10^8^ CFU/g *Bacillus amyloliquefaciens* SC06; SC06 + DQ, control diet containing 1 × 10^8^ CFU/g *Bacillus amyloliquefaciens* SC06 plus diquat injection.

Figure 3A showed that the DQ treatment significantly increased the gene expressions of *GRP78* (*p* < 0.01), *PERK* (*p* < 0.01), *eIF2α* (*p* < 0.01), *IRE1-α* (*p* = 0.03), *CHOP* (*p* < 0.01), *ERO1α* (*p* = 0.01) and *ATF4* (*p* < 0.01). The SC06 supplementation significantly decreased the gene expressions of *GRP78* (*p* < 0.01), *PERK* (*p* < 0.01), *eIF2α* (*p* = 0.01), *ATF6* (*p* = 0.03), *IRE1-α* (*p* < 0.01), *CHOP* (*p* < 0.01), *ERO1α* (*p* < 0.01) and *ATF4* (*p* < 0.01). Moreover, SC06 × DQ increased the gene expressions of *GRP78* (*p* < 0.01), *PERK* (*p* < 0.01), *eIF2α* (*p* < 0.01), *ATF6* (*p* = 0.03), *IRE1-α* (*p* = 0.01), *CHOP* (*p* < 0.01), *ERO1α* (*p* < 0.01) and *ATF4* (*p* < 0.01) in DQ-treated piglets. Western blotting results further indicated that the expressions of GRP78 (*p* < 0.01), ATF4 (*p* = 0.03) and p-PERK/PERK (*p* < 0.01) were significantly up-regulated. The supplementation of SC06 down-regulated the expressions of GRP78, ATF4, PERK and p-PERK/PERK (*p* < 0.01). Besides, SC06 × DQ also decreased the expressions of GRP78 and p-PERK/PERK (*p* < 0.01) in DQ-injected piglets (Figure 3B).

### 3.5 Transcription changes of weaned piglets with SC06 supplementation

**Figure 4:**
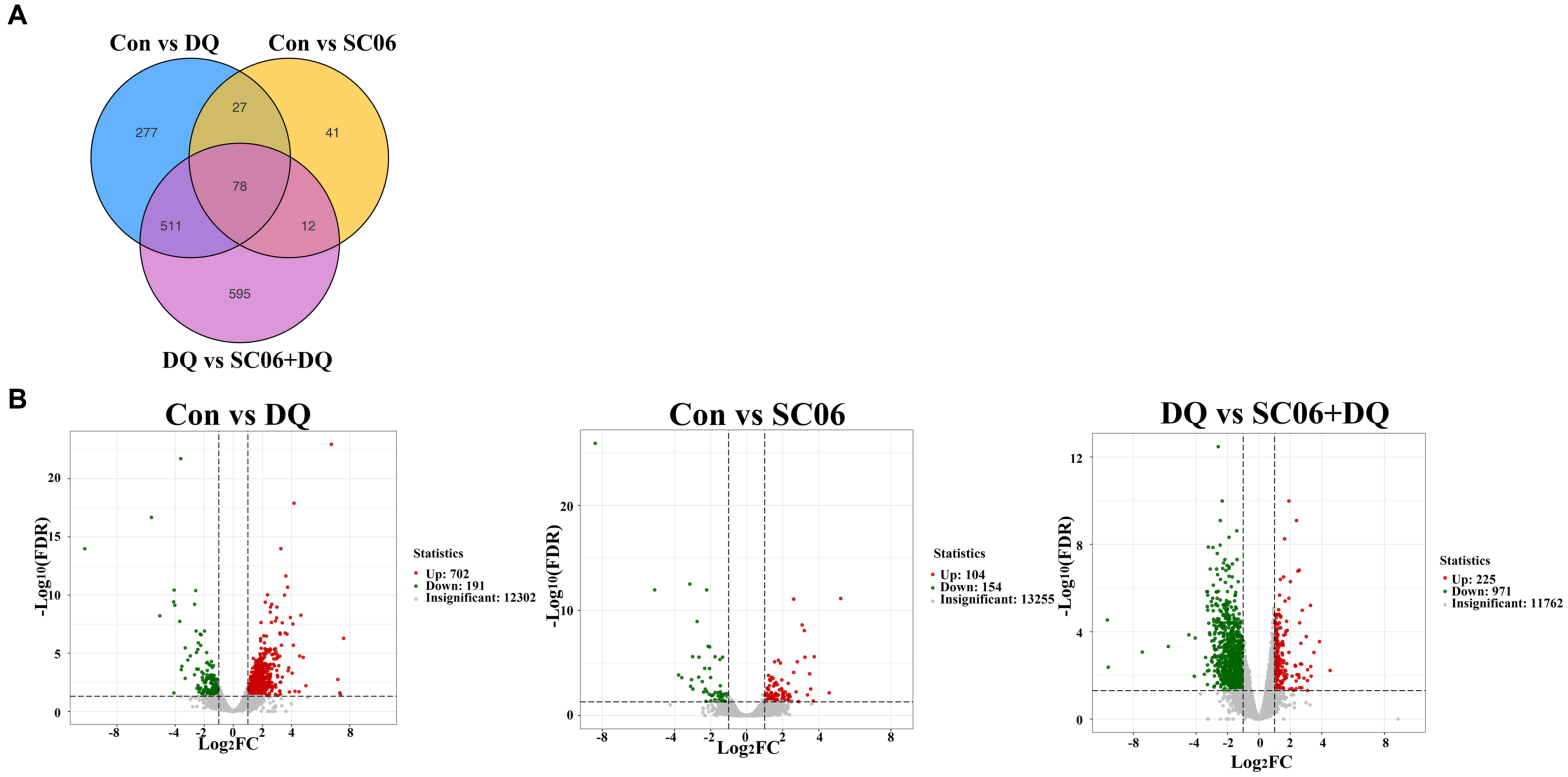
Differentially expressed genes in the ileal mucosal of weaned piglets. **(A)** Venn diagrams between different comparation groups. **(B)** Volcano plot between groups. *n* = 3. Con, control diet; DQ, control diet plus diquat injection; SC06, control diet containing 1 × 10^8^ CFU/g *Bacillus amyloliquefaciens* SC06; SC06 + DQ, control diet containing 1 × 10^8^ CFU/g *Bacillus amyloliquefaciens* SC06 plus diquat injection.

A total of 105 common genes were found between the Con vs. DQ and Con vs. SC06 groups, 589 common genes were found between the Con vs. DQ and DQ vs. SC06 + DQ groups, 90 common genes were found between the Con vs. SC06 and DQ vs. SC06 + DQ groups (Figure 4A). Moreover, compared with the Con group, 702 genes were upregulated (such as *ZNF473*, *GGT5*, *FBXO39*, etc.) and 191 genes were downregulated (such as *GPT2*, *NR1D1*, *SOD3*, etc.) in the DQ group (*p* < 0.05); 104 genes were upregulated (such as *SLC25A37*, *CLCN1*, *TRUB1*, etc.) and 154 genes were downregulated (such as *DPYS*, *APOA4*, *SLC25A20,* etc.) in the SC06 group (*p* < 0.05). Compared with the DQ group, 225 genes were upregulated (such as *ZNF473*, *PRDX6*, *GPT2*, etc.) and 971 genes were downregulated (such as *GGT5*, *OGT*, *CTSK*, *SLC25A37*, etc.) in the SC06 + DQ group (*p* < 0.05) (Figure 4B and Supplementary Tables S2–S4).

### 3.6 Functional analysis of the DEGs

**Figure 5:**
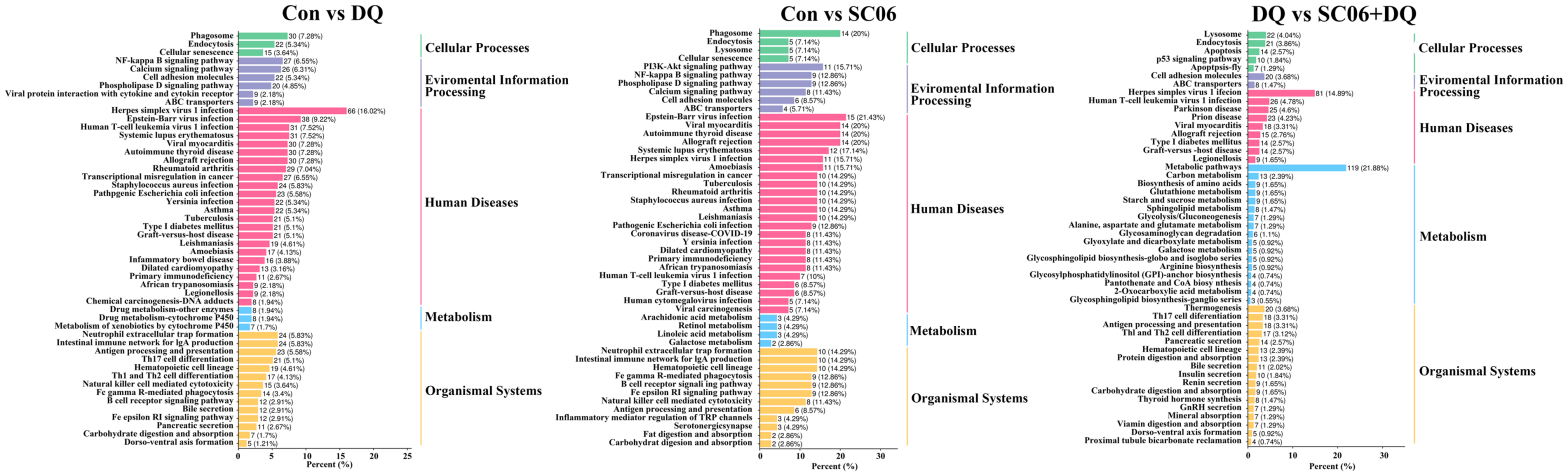
KEGG pathway analysis of genes in the ileal mucosal of weaned piglets. *n* = 3. Con, control diet; DQ, control diet plus diquat injection; SC06, control diet containing 1 × 10^8^ CFU/g *Bacillus amyloliquefaciens* SC06; SC06 + DQ, control diet containing 1 × 10^8^ CFU/g *Bacillus amyloliquefaciens* SC06 plus diquat injection.

KEGG analysis showed that the enriched pathways of DEGs between the DQ group and the Con group included NF-κB signaling pathway, Intestinal immune network for IgA production, Phagosome, etc. The enriched pathways of DEGs between the SC06 group and the Con group included PI3K-Akt signaling pathway, NF-κB signaling pathway, Phagosome, etc. The enriched pathways of DEGs between the SC06 + DQ group and the DQ group included Arginine biosynthesis, Protein digestion and absorption, Glutathione metabolism, etc. (Figure 5).

### 3.7 Validation of the expressions of DEGs by RT-qPCR

**Figure 6:**
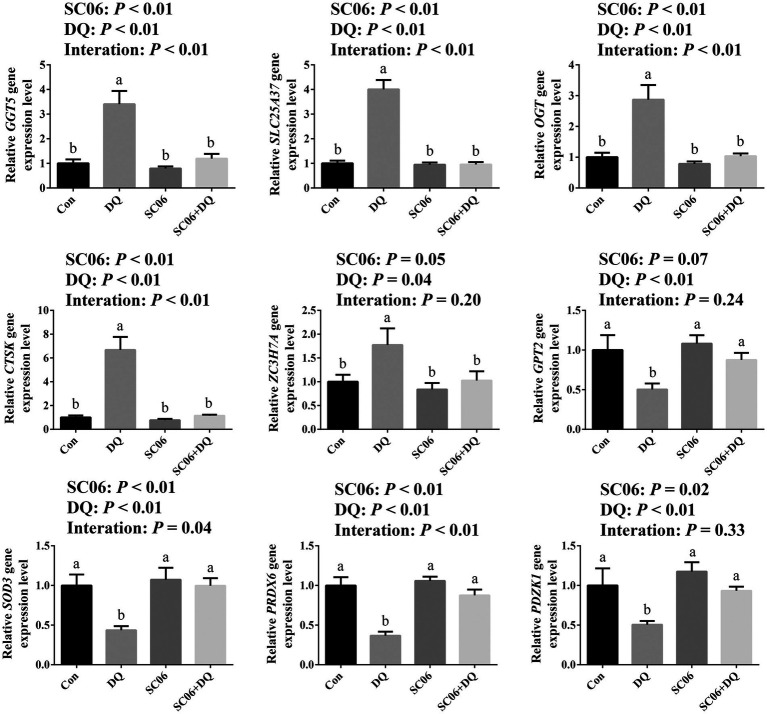
Validation of DEGs by real-time quantitative PCR. ^a,b,c^Mean value within a row with no common superscript differ significantly (*p* < 0.05), *n* = 6. *GGT5*, gamma-glutamyltransferase 5; *SLC25A37*, solute carrier family 25 member 37; *OGT*, EGF domain specific O-linked N-acetylglucosamine transferase; *CTSK*, cathepsin K; *ZC3H7A*, zinc finger CCCH-type containing 7A; *GPT2*, glutamic pyruvic transaminase 2; *SOD3*, superoxide dismutase 3; *PRDX6*, peroxiredoxin 6; *PDZK1*, PDZ domain containing 1; Con, control diet; DQ, control diet plus diquat injection; SC06, control diet containing 1 × 10^8^ CFU/g *Bacillus amyloliquefaciens* SC06; SC06 + DQ, control diet containing 1 × 10^8^ CFU/g *Bacillus amyloliquefaciens* SC06 plus diquat injection.

The DEGs that related to OS and ER stress were selected for validation by RT-qPCR. Results showed that the DQ treatment increased the expressions of *GGT5, SLC25A37, OGT, CTSK* (*p* < 0.01) and *ZC3H7A* (*p* = 0.04), and decreased the expressions of *GPT2, SOD3, PRDX6* and *PDZK1* (*p* < 0.01). The SC06 supplementation decreased the expressions of *GGT5, SLC25A37, OGT, CTSK* (*p* < 0.01), *ZC3H7A* (*p* = 0.05), and increased the expressions of *SOD3*, *PRDX6* (*p* < 0.01) and *PDZK1* (*p* = 0.02). Moreover, SC06 × DQ reversed the changes in *GGT5, SLC25A37, OGT*, *CTSK*, *PRDX6* (*p* < 0.01) and *SOD3* (*p* = 0.04) in DQ-treated piglets (Figure 6).

### 3.8 Ileal mucosal microbiota changes of weaned piglets with SC06 supplementation

**Figure 7:**
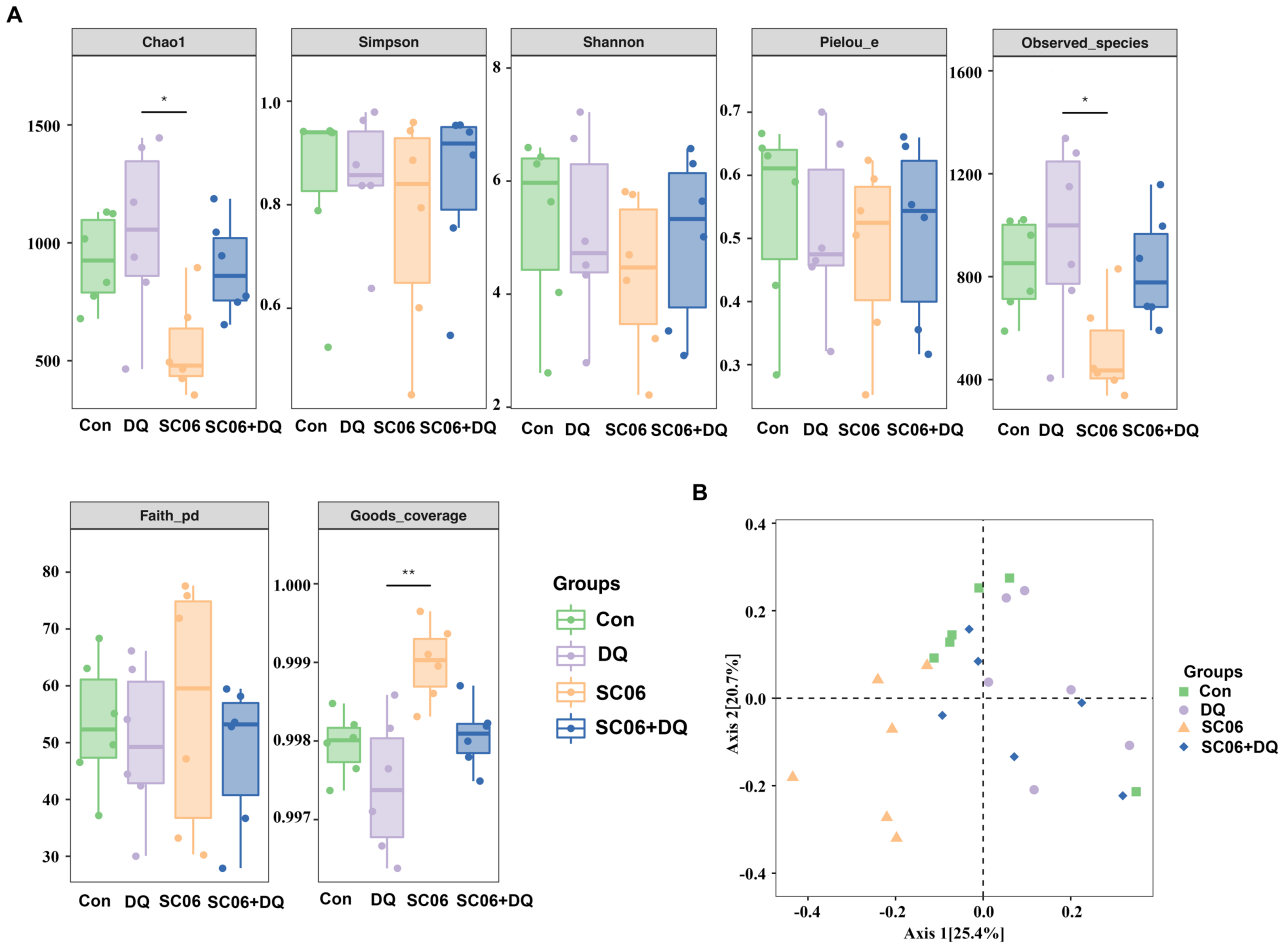
Effects of SC06 on the *α*- and *β*-diversity of ileal mucosal microbiota. **(A)** *α*-diversity. **(B)** *β*-diversity. *n* = 6. Con, control diet; DQ, control diet plus diquat injection; SC06, control diet containing 1 × 10^8^ CFU/g *Bacillus amyloliquefaciens* SC06; SC06 + DQ, control diet containing 1 × 10^8^ CFU/g *Bacillus amyloliquefaciens* SC06 plus diquat injection.

**Figure 8:**
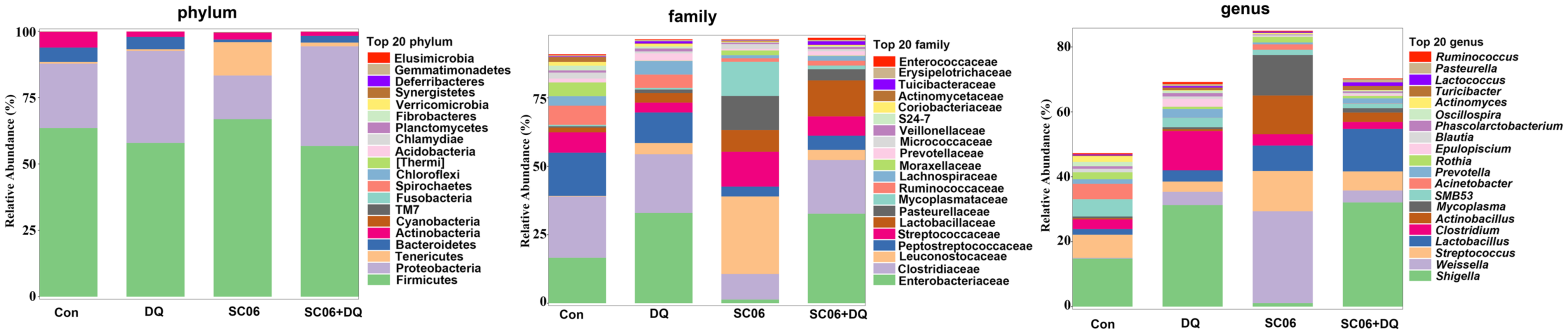
The top 20 phylum, family and genus of ileal mucosal microbiota. *n* = 6. Con, control diet; DQ, control diet plus diquat injection; SC06, control diet containing 1 × 10^8^ CFU/g *Bacillus amyloliquefaciens* SC06; SC06 + DQ, control diet containing 1 × 10^8^ CFU/g *Bacillus amyloliquefaciens* SC06 plus diquat injection.

The *α*-diversity indices, including Chao1, Simpson, Shannon, Pielou_e, Observed_species, Faith_pd and Goods_coverage were not significantly different between the Con and DQ groups, Con and SC06 groups, as well as DQ and SC06 + DQ groups (Figure 7A). Moreover, there was no difference in the *β*-diversity among groups (Figure 7B). However, the ileal mucosal microbiota composition was significantly influenced by different treatments. At the phylum level, the DQ treatment decreased the abundance of Acidobacteria compared with the Con group (*p* < 0.05). As for the top 20 family, we found that in comparison with the Con group, the DQ treatment increased the abundance of Turicibacteraceae (*p* < 0.05); the SC06 treatment decreased the abundance of Ruminococcaceae (*p* < 0.05). Compared with the DQ group, the SC06 + DQ treatment increased the abundance of Pasteurellaceae (*p* < 0.05). As for the top 20 genus, compared with the Con group, the DQ treatment increased the abundances of *Clostridium* and *Turicibacter* (*p* < 0.05); the SC06 treatment increased the abundance of *Lactobacillus* (*p* < 0.05). Compared with the DQ group, the SC06 + DQ treatment decreased the abundance of *Clostridium* (*p* < 0.01) and increased the abundance of *Actinobacillus* (*p* < 0.05) (Figure 8 and Supplementary Table S5).

### 3.9 Functional analysis of the ileal mucosal microbiota

**Figure 9:**
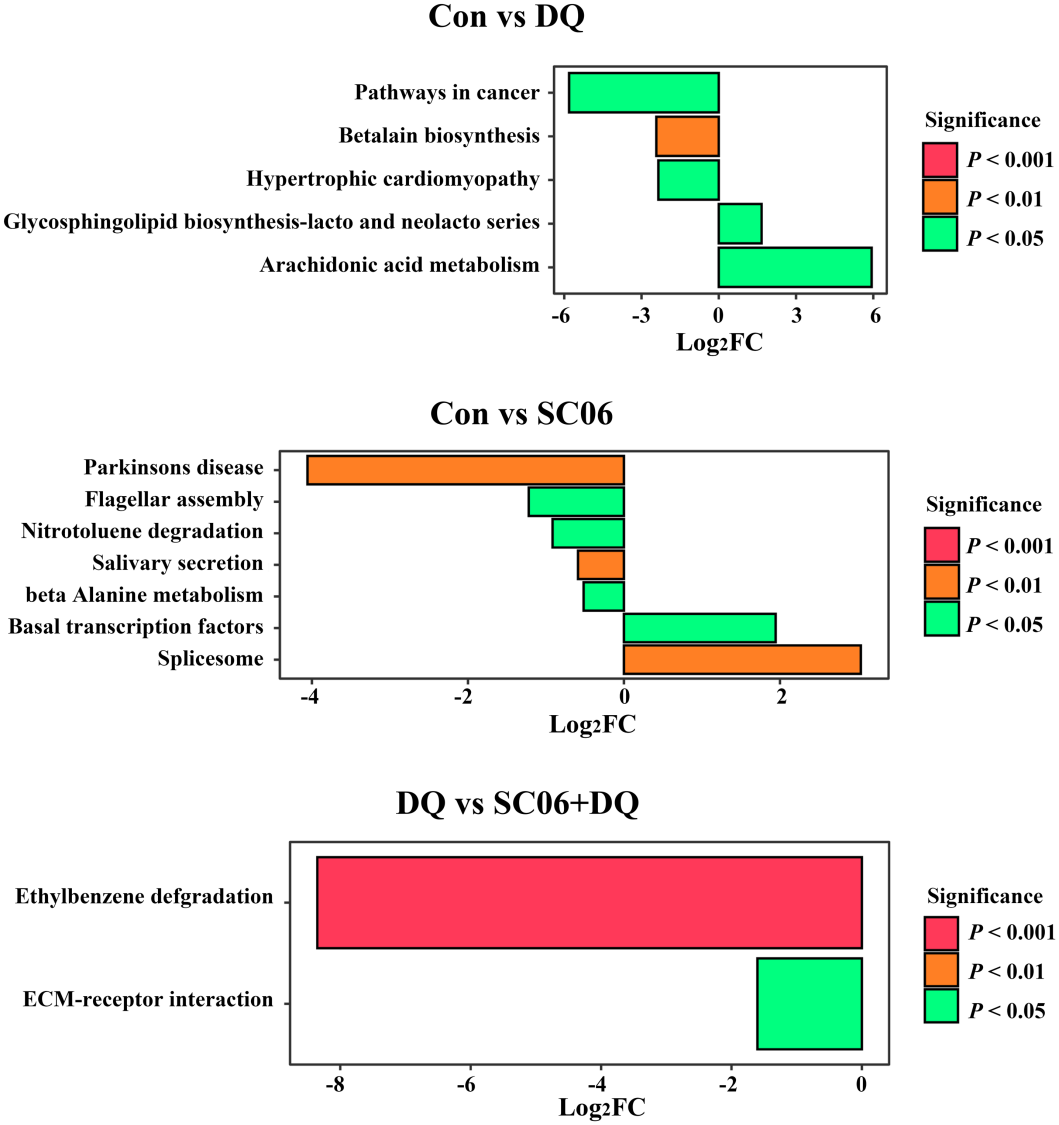
Functional prediction of ileal mucosal microbiota. *n* = 6. Con, control diet; DQ, control diet plus diquat injection; SC06, control diet containing 1 × 10^8^ CFU/g *Bacillus amyloliquefaciens* SC06; SC06 + DQ, control diet containing 1 × 10^8^ CFU/g *Bacillus amyloliquefaciens* SC06 plus diquat injection.

Compared with the Con group, piglets in the DQ group had lower levels of microbial genes associated with Arachidonic acid metabolism (*p* < 0.05) and Glycosphingolipid biosynthesis-lacto and neolacto series (*p* < 0.05), and higher levels of microbial genes related to Betalain biosynthesis (*p* < 0.01), Pathways in cancer (*p* < 0.05) and Hypertrophic cardiomyopathy (*p* < 0.05); piglets in the SC06 group had lower levels of microbial genes associated with Spliceosome (*p* < 0.01) and Basal transcription factors (*p* < 0.05), and higher levels of microbial genes related to Beta-alanine metabolism (*p* < 0.05), Salivary secretion (*p* < 0.01), Nitrotoluene degradation (*p* < 0.05), Flagellar assembly (*p* < 0.05) and Parkinsons disease (*p* < 0.01). Moreover, compared with the DQ group, piglets in the SC06 + DQ group had higher levels of microbial genes related to ECM-receptor interaction (*p* < 0.05) and Ethylbenzene degradation (*p* < 0.01) (Figure 9).

### 3.10 Correlation analysis between ER stress-related genes and bacterial genus abundance

**Figure 10:**
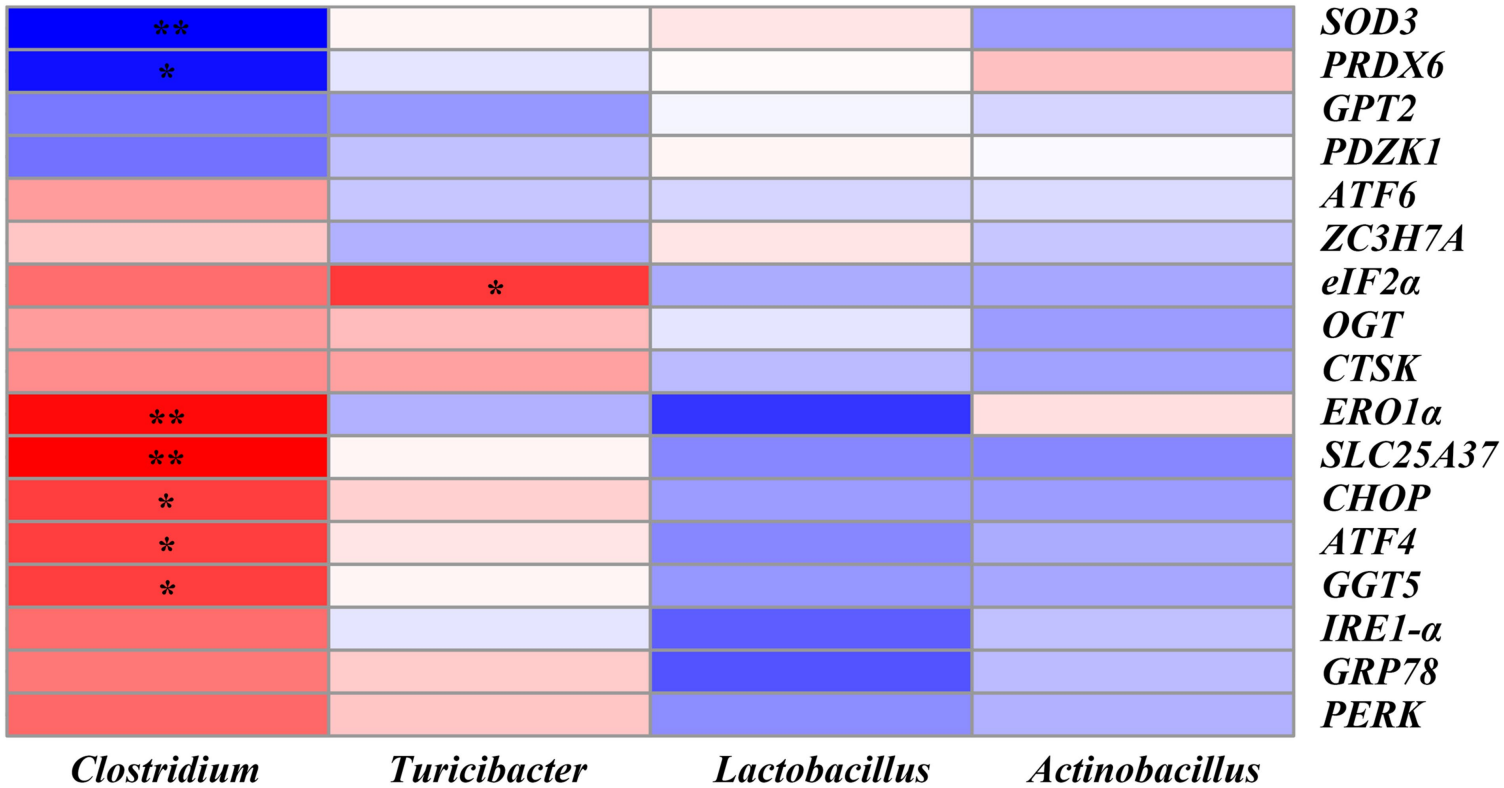
Heatmap of Pearson’s correlation analysis between ER stress-related genes and the top 20 microbiota genera. * = *p* < 0.05, ** = *p* < 0.01, *n* = 6. *SOD3*, superoxide dismutase 3; *PRDX6*, peroxiredoxin 6; *GPT2*, glutamic pyruvic transaminase 2; *PDZK1*, PDZ domain containing 1; *ATF6*, activating transcription factor 6; *ZC3H7A*, zinc finger CCCH-type containing 7A; *eIF2α*, phosphorylation of eukaryotic initiation factor-2*α*; *OGT*, EGF domain specific O-linked N-acetylglucosamine transferase; *CTSK*, cathepsin K; *ERO1α*, endoplasmic reticulum oxidoreductin 1*α*; *SLC25A37*, solute carrier family 25 member 37; *CHOP*, C/EBP homologous protein; *ATF4*, activating transcription factor 4; *GGT5*, gamma-glutamyltransferase 5; *IRE1-α*, inositol-requiring enzyme 1*α*; *GRP78*, glucose-regulated protein 78; *PERK*, protein kinase RNA-like endoplasmic reticulum kinase.

The correlation between the ER stress-related genes and the significantly altered top 20 genera was shown in Figure 10. We found that *SLC25A37* (*r* = 0.62, *p* < 0.01), *ERO1α* (*r* = 0.58, *p* < 0.01), *CHOP* (*r* = 0.48, *p* < 0.05), *ATF4* (*r* = 0.47, *p* < 0.05) and *GGT5* (*r* = 0.47, *p* < 0.05) were positively correlated with the abundance of *Clostridium*. *SOD3* (*r* = −0.52, *p* < 0.01) and *PRDX6* (*r* = −0.48, *p* < 0.05) were significantly negatively correlated with *Clostridium*. Moreover, *eIF2α* was positively correlated with the abundance of *Turicibacter* (*r* = 0.48, *p* < 0.05) (Figure 10 and Supplementary Table S6).

## 4 Discussion

In the production of pig farming, various factors can induce intestinal OS and impair intestinal barrier function, leading to economic losses. Probiotics are well-known in regulating growth performance and gut health. In the present study, the DQ injection impaired the growth performance, ileal morphology and barrier function. Supplementation of SC06 improved the morphology, increased ADG and the expressions of tight junctions and decreased the permeability of ileum. Moreover, SC06 × DQ improved ADG, ileal morphology and barrier functions in DQ-treated piglets, which were similar to the findings of previous studies on the beneficial effects of probiotics in pig growth and intestinal health (Wang et al., 2018; Sun et al., 2023).

Excessive amounts of ROS can induce OS, causing lipid peroxidation and augmenting MDA levels. Here the elevated ileal ROS and MDA levels in DQ-treated piglets indicated the increased OS. On the contrary, the SC06 supplementation and SC06 × DQ decreased the ROS and MDA levels. The major antioxidant defense machineries for most living organisms are composed of biological antioxidants and antioxidant enzymes, such as GSH-Px, CAT and SOD. Here we found that the SC06 supplementation reversed the decreased activities of GSH-Px, CAT and SOD in DQ-treated piglets. These results were consistent with the findings showing that dietary supplementation of probiotics enhanced the antioxidant capacity of piglets (Wang et al., 2017a; Yu et al., 2022).

OS is associated with ER stress (Doyle et al., 2011). Recent studies have also shown that ER stress plays important roles in intestinal barrier function and homeostasis (Wan et al., 2022; Tan et al., 2023). Therefore, the ER stress-related parameters were further detected. We found that the ER stress-related genes, including *GRP78*, *PERK*, *eIF2α*, *ATF4*, *ATF6*, *IRE1-α*, *CHOP* and *ERO1α* were increased in the DQ-treated piglets, implying the elevation of ER stress. However, the SC06 supplementation or the SC06 × DQ decreased the expressions of the above genes. Western blotting further confirmed the decreased ER stress in oxidatively-stressed piglets receiving SC06.

In recent years, with the rapid development of next-generation sequencing, RNA-sequencing (RNA-seq) has become one of the important means to study genes function and screen new genes and has been widely used in animal husbandry. By using ileal transcriptome, Tang et al. (2022) identified the key differentially expressed genes (DEG) involved in the chronic heat stress-induced ER stress in growing piglets. In the study by Cui et al. (2021), transcriptome sequencing analysis revealed that OS activated the ER stress response/UPR but inhibited glutathione metabolism based on the screened DEGs. Here, ileal transcriptome demonstrated that the DEGs between the DQ and Con groups mainly included *GPT2*, *GGT5*, *SOD3*, etc. The DEGs between the SC06 and Con groups mainly included *SLC25A37*, *TRUB1*, *APOA4*, *SLC25A20*, etc. The DEGs between the SC06 + DQ and DQ groups mainly included *PRDX6*, *GGT5*, *OGT*, *CTSK*, etc. These DEGs are reported to be associated with OS or ER stress. *GGT5* is confirmed to regulate immunity and OS (Li et al., 2016). The change in *GPT2* in murine mammary cancer cells expression was accompanied by the increase of the ATF4 (Kiesel et al., 2022). SOD3 is also related to OS (Wert et al., 2018). Reduction of SOD3 was found in ursodeoxycholic acid (an ER stress inhibitor)-treated diabetic mice (Cao et al., 2016). Moreover, in the study by Bie et al. (2023), serum RNA sequencing identified *SLC25A37* is the key gene that associated with ER stress. OGT is a unique glycosyltransferase involved in metabolic reprogramming. Xu et al. (2017) indicated that OGT promotes fatty liver-associated liver cancer through inducing palmitic acid and activating ER stress. Besides, two ER stress inducers, tunicamycin and thapsigargin, induced expressions of GRP78, IRE-1, TRAP, and CTSK at both protein and mRNA levels, indicating the CTSK is also related to ER stress (Wang et al., 2011). MacLeod et al. (2019) suggested that proximal interactors of ZC3H7A were enriched in biological processes related to translation regulation, mRNA processing, cytoplasmic stress granule, and p53-mediated DNA damage signaling. PRDX6 is a cytoprotective protein by regulating intracellular ROS. Fatma et al. (2011) demonstrated that *PRDX6* deficiency in cells evoked ER stress, evidenced by increased expression or activation of proapoptotic factors, CHOP, ATF4, PERK, IRE-*α* and eIF2-*α* and by increased caspases 3 and 12 processing. PDZK1 was reported to regulate cellular apoptosis (Yu et al., 2018a), but their study also showed that leukocyte PDZK1 deficiency had no significant effect on macrophage ER stress in atherosclerotic plaques of low density lipoprotein receptor deficient mice (Yu et al., 2018b). Taken together, the alterations of the above DEGs may be associated with the changes of OS and ER stress in different groups.

Further, KEGG analysis showed that the DEGs between the DQ and Con groups were mostly enriched in the NF-κB signaling pathway, Intestinal immune network for IgA production, Phagosome, etc. The first eukaryotic transcription factor shown to respond directly to OS was NF-κB (Schreck et al., 1991). During inflammation, ROS can mediate the activation of NF-κB, and the subsequent expression of inflammatory cytokines (Srivastava et al., 2011). In recent decades, a crosstalk between NF-κB and ER stress has been found (Li et al., 2022). ER stress can activate NF-κB by integrating functions of basal IKK activity, IRE1 and PERK (Tam et al., 2012). Therefore, the enriched NF-κB signaling pathway was in accordance with the increased OS and ER stress in the DQ group. Moreover, the DEGs between the SC06 and Con groups were mostly enriched in PI3K-Akt signaling pathway, NF-κB signaling pathway, Phagosome, etc. PI3K-Akt signaling pathway can activate Nrf2 to ameliorate OS (Wen et al., 2018). Research showed that PI3K-Akt inactivation could induce CHOP expression in ER stressed cells (Hyoda et al., 2006). Thus, the enriched PI3K-Akt signaling pathway may imply the attenuated OS and ER stress by the SC06 treatment. Additionally, the DEGs between the SC06 + DQ and DQ groups were mostly enriched in Arginine biosynthesis, Protein digestion and absorption, Glutathione metabolism, etc. Glutathione metabolism is well-known in redox regulation (Yuan et al., 2023a). Arginine, a basic amino acid, serves as an essential precursor for the synthesis of biologically important molecules such as protein, ornithine, proline, polyamines, creatine, NO and agmatine (Wu and Morris, 1998). The beneficial effects of arginine, including antioxidation were reported (Saleh and El-Demerdash, 2005). Furthermore, García-Navas et al. (2012) suggested that depletion of L-arginine induces autophagy as a cytoprotective response to endoplasmic reticulum stress in human T lymphocytes. Hence, the enriched Arginine biosynthesis and Glutathione metabolism suggested the decrease of OS and ER stress in SC06 + DQ group compared with the DQ group.

In addition, gut microbiota protects their hosts from pathogens through competitive exclusion. When the gut microbiota is abnormal, harmful bacteria will overproliferate and induce the endotoxin in blood, causing significant OS (Wang et al., 2017a). Although numerous studies explored the cecal microbiota or fecal microbiota, the studies on intestinal mucosal microbiota are relatively little. Several research have proved that there are different in composition and diversity of microbiota in the lumen and mucosa (Wu et al., 2020). Moreover, together with intestinal mucosa, mucin, secretory immunoglobulin A and intestinal mucosal microbiota are vital in blocking and delaying the translocation and infection of various pathogens (Yap and Mariño, 2018). In the present study, altered ileal mucosal microbiota composition was observed among groups. As for the phylum, and top 20 family and genus, compared with the Con group, DQ decreased the Acidobacteria abundance and increased the abundances of Turicibacteraceae, *Clostridium* and *Turicibacter*; SC06 decreased Ruminococcaceae abundance and increased *Lactobacillus* abundance. Compared with the DQ group, SC06 + DQ decreased the *Clostridium* abundance and increased the abundances of *Pasteurellaceae* and *Actinobacillus*. *Turicibacter* belongs to the Firmicutes phylum. *Turicibacter* was associated with rat models of trinitrobenzene sulfonic acid-induced colitis and diabetes, might also affect intestinal health and cause various diseases, including diabetes, inflammation (Jones-Hall et al., 2015). *Clostridium* genera are mostly known as pathogenic microorganisms (Samul et al., 2013). Most of the studies conducted during the weaning transition have reported an increase of *Clostridium* (Gresse et al., 2017). *Acinetobacter* are dominant genera in the small intestine and considered to take part in digestion process (Zhao et al., 2015). Some species of *Acinetobacter* have been applied in the degradation of lignin and amino acids (Adegoke et al., 2012). *Lactobacillus* spp. are well-known probiotics and have beneficial role in growth promotion, antioxidation and anti-inflammation (Valeriano et al., 2017). Taken together, the increased abundances of *Clostridium* and *Turicibacter* indicated the increased ileal inflammation by the DQ treatment. The increased *Lactobacillus* abundance in the SC06 group, decreased *Clostridium* abundance and increased *Actinobacillus* abundance in the SC06 + DQ group, were related to the improved ileal health and digestive capacity of piglets.

Moreover, our KEGG results of ileal mucosal microbiota indicate that some diseases-related pathways, including the Hypertrophic cardiomyopathy and Pathways in cancer were upregulated in the DQ group compared with the Con group. Moreover, ECM-receptor interaction and Ethylbenzene degradation pathways were upregulated in the SC06 + DQ group compared with the DQ group. ECM-receptor interactions are pathways that maintain cell and tissue structure and function. ECM-receptors mediate ECM protein interactions and promote the macromolecules formation (Mo et al., 2023). Ethylbenzene is a toxic aromatic organic compound. The treatment of SC06 + DQ significantly enriched microbial functional genes related to the degradation of the ethylbenzene, suggesting that the microbes may have a better ability to degrade toxic organic compounds and maintain homeostasis of the gut environment. Similar result was found by Liu et al. (2021), in which the gut microbial genes of chickens treated with multi-enzyme were also enriched in the Ethylbenzene degradation pathway.

In addition, correlation analysis further showed that there were significant correlations between the ER stress-related genes and the abundance of microbial genus. For example, *SLC25A37* and *ERO1α* were positively correlated with, while *SOD3* was negatively correlated with the abundance of *Clostridium*. *eIF2α* was positively correlated with the abundance of *Turicibacter.* Since *Clostridium* was significantly reduced in the SC06 + DQ group, it may be the key bacterium responsible for the alleviation of OS by SC06 in weaned piglets.

## 5 Conclusion

In summary, our data show a significant improvement in growth performance, ileal morphology, barrier function, antioxidant and anti-ER stress capacity of dietary SC06 supplementation in DQ-treated weaned piglets. By taking advantages of microbiome and transcriptome analyses, we found significant reshaping of ileal mucosal bacteria (including *Clostridium* and *Turicibacter*) with SC06 supplementation, along with the changes in ileal genes (including *SLC25A37*, *ERO1α, SOD3* and *eIF2α*).

## Data availability statement

The datasets presented in this study can be found in online repositories. The names of the repository/repositories and accession number(s) can be found at: NCBI - PRJNA1021385 and PRJNA1012569.

## Ethics statement

The animal study was reviewed and approved by the Animal Care and Use Committee of Qingdao Agricultural University (protocol number 20221125374). The study was conducted in accordance with the local legislation and institutional requirements.

## Author contributions

JY: Writing – original draft, Writing – review & editing. HM: Formal analysis, Writing – original draft. YL: Formal analysis, Writing – original draft. LW: Data curation, Writing – original draft. QZ: Data curation, Writing – original draft. ZW: Data curation, Writing – original draft. HL: Funding acquisition, Writing – original draft. KZ: Funding acquisition, Writing – original draft. JZ: Funding acquisition, Writing – original draft. WL: Conceptualization, Writing – original draft, Writing – review & editing. YW: Conceptualization, Writing – original draft, Writing – review & editing.

## Footnotes

^1^ https://ftp.ncbi.nlm.nih.gov/genomes/refseq/vertebrate_mammalian/Sus_scrofa/latest_assembly_versions/GCF_000003025.6_Sscrofa11.1/

^2^ https://www.kegg.jp/

^3^ https://www.biodeep.cn/home
