# Supplementary material for: Bacillus amyloliquefaciens attenuates the intestinal permeability, oxidative stress and endoplasmic reticulum stress: transcriptome and microbiome analyses in weaned piglets
Source: Front Microbiol. 2024 May 13;15:1362487. doi: 10.3389/fmicb.2024.1362487 (PMC11131103; doi:10.3389/fmicb.2024.1362487)
Supplement: Supplementary file 1 [file Data_Sheet_1.docx]

Supplementary Material

**Supplementary Table 1:** Primers used for real time-PCR

| Gene | Forward Primer sequence（5'-3') | Reverse Primer sequence（5'-3') |
| --- | --- | --- |
| *β-actin* | CCCAAAGCCAACCGTGAGAA | CCACGTACATGGCTGGGGTG |
| *GAPDH* | CAGCAATGCCTCCTGTACCA | CCACGATGCCGAAGTTGTC |
| *GRP78* | CGGAGGAGGAGGACAAGAAGGAG | ATATGACGGCGTGATGCGGTTG |
| *PERK* | TGGGTTGTCGCCAATGGGATAATG | ATTCCACTTCTCGTTGCCACTGC |
| *eIF2α* | TGATGGACCTCATGCAGCTTTAGC | ACGCTCCTGTCTTGTCAACTTCTG |
| *ATF4* | AGGAGTTCGACTTGGATGCCCTG | AGTGATATCCACTTCACTGCCCAG |
| *ATF6* | GCTCCTCCGTTCCTCCTTACCTC | CTGACAACATGGGCTGCCTCTG |
| *IRE1-α* | CGGGCCATGAGAAATAAGAA | GTAGGTGTGTGCCAGGAGGT |
| *CHOP* | TCTGGCTTGGCTGACTGAGGAG | CCGTTTCCTGGGTCTTCTTTGGTC |
| *ERO1α* | TGAGGAAACGCAGAAGGCTGTTC | TCTGGTCCCTTGTAACCCGTGTAG |
| *GPT2* | GGGGCTACAGGTTTCTCTGG | TGGTTTCTTGATACCCCGCT |
| *SOD3* | GCTGCTCTGTGCTTACCTGCTC | CTGCGTCAACTCCTGCCAGATC |
| *GGT5* | GCTCTGTTCTTGCTCACCCTG | CTCTTGCCCAATCCCACTCT |
| *SLC25A37* | AGTGTCTACGGAGCCCTCAA | ATACTCCCAGCTATCCCGTTG |
| *PRDX6* | AGACCCAGCAGAAAAGGACG | CGCTGTCTCCATTCTTCCAGT |
| *OGT* | CCTGTAACTGTTGCCGCCT | ACGTTTCGTTGGTTCTGTGCT |
| *CTSK* | ACTGGATAATTAAAAACAGCTGGGG | AGGAAAGACACACCAGTTCCC |
| *PDZK1* | GGAGTGAATGTGCTGGATGA | GCCTTCTTTCCACAGACTAGG |
| *ZC3H7A* | ACATGTCCAGTGTGTCCGAG | CCAGTCACGCTCCCGATAAA |

| **NCBI Gene ID** | **Gene name** | **up/down** | **Log_2_FC** | ***Q* value** |
| --- | --- | --- | --- | --- |
| 106504168 | *FBXO39* | up | 2.34 | <0.01 |
| 110261049 | *RDH13* | up | 2.19 | <0.01 |
| 100520981 | *NFKBIZ* | up | 2.56 | <0.01 |
| 100517358 | *PIWIL4* | up | 3.92 | <0.01 |
| 553951 | *CCL20* | up | 2.97 | <0.01 |
| 110261148 | *ZC3H12A* | up | 1.90 | <0.01 |
| 396880 | *CXCL8* | up | 3.36 | <0.01 |
| 100626104 | *SDR9C7* | up | 3.56 | <0.01 |
| 399688 | *DMP1* | up | 3.20 | <0.01 |
| 100524202 | *ZNF473* | up | 2.09 | <0.01 |
| 100624598 | *MEIOB* | up | 2.27 | <0.01 |
| 100157072 | *ALDH5A1* | up | 1.87 | <0.01 |
| 100526033 | *FNBP4* | up | 1.87 | <0.01 |
| 100627364 | *CCDC191* | up | 2.14 | <0.01 |
| 100525660 | *WSB1* | up | 1.91 | <0.01 |
| 100627131 | *SLC9C1* | up | 2.53 | <0.01 |
| 100628093 | *TOP1MT* | up | 2.81 | <0.01 |
| 100511190 | *CCDC138* | up | 2.24 | <0.01 |
| 100524368 | *DECR2* | up | 2.24 | <0.01 |
| 100515310 | *WDR97* | up | 2.92 | <0.01 |
| 100154429 | *LSMEM2* | up | 2.46 | <0.01 |
| 102164483 | *ALS2CL* | up | 2.06 | <0.01 |
| 100157523 | *GGT5* | up | 2.24 | <0.01 |
| 100169932 | *TRPM7* | up | 1.83 | <0.01 |
| 100525327 | *ANKRD24* | up | 2.71 | <0.01 |
| 100510970 | *CC2D2A* | up | 2.66 | <0.01 |
| 100521318 | *GPT2* | down | -2.28 | <0.01 |
| 641342 | *NR1D1* | down | -2.21 | <0.01 |
| 100621722 | *FBLN2* | down | -3.31 | <0.01 |
| 780439 | *SOD3* | down | -3.31 | <0.01 |

**Supplementary Table 2:** Top 30 DEGs between the DQ and Con groups

DEGs = differentially expressed known genes, Con = control diet, DQ = control diet plus diquat injection, FC = fold change, n = 3 for each group.

**Supplementary Table 3:** Top 30 DEGs between the SC06 and Con groups

| **NCBI Gene ID** | **Gene name** | **up/down** | **Log_2_FC** | ***Q* value** |
| --- | --- | --- | --- | --- |
| 106504168 | *FBXO39* | up | 1.76 | <0.01 |
| 100520812 | *CLCN1* | up | 1.55 | <0.01 |
| 100151860 | *TRUB1* | up | 2.61 | <0.01 |
| 100625807 | *LCT* | up | 3.49 | <0.01 |
| 100621580 | *DDX59* | up | 1.21 | <0.01 |
| 100514004 | *CARD14* | up | 1.39 | <0.01 |
| 110260406 | *EPPK1* | up | 1.47 | <0.01 |
| 100515587 | *NEMP2* | up | 1.33 | <0.01 |
| 100518366 | *DQX1* | up | 1.26 | <0.01 |
| 106505668 | *TTLL3* | up | 1.50 | <0.01 |
| 100516679 | *BEST4* | up | 1.14 | <0.01 |
| 102164483 | *ALS2CL* | up | 1.17 | <0.01 |
| 100153622 | *SLC25A37* | up | 1.22 | <0.01 |
| 100519954 | *WDR35* | up | 1.82 | <0.01 |
| 102161626 | *ZNF132* | up | 2.75 | <0.01 |
| 100154080 | *REC114* | up | 2.00 | <0.01 |
| 100156500 | *DPYS* | down | -5.10 | <0.01 |
| 397681 | *APOA4* | down | -2.22 | <0.01 |
| 403108 | *CYP2C34* | down | -2.75 | <0.01 |
| 100512615 | *ADH1C* | down | -2.05 | <0.01 |
| 100134959 | *G6PC* | down | -3.00 | <0.01 |
| 100626412 | *RAB30* | down | -1.75 | <0.01 |
| 100524459 | *SLC25A20* | down | -1.33 | <0.01 |
| 100522979 | *CREB3L3* | down | -1.48 | <0.01 |
| 100154266 | *ABHD4* | down | -2.06 | <0.01 |
| 403110 | *CYP2C36* | down | -3.78 | <0.01 |
| 100521495 | *GTSF1* | down | -3.60 | <0.01 |
| 100134962 | *PHYH* | down | -2.29 | <0.01 |
| 397393 | *CAPN2* | down | -1.45 | <0.01 |
| 100154412 | *AP3B2* | down | -2.39 | <0.01 |

DEGs = differentially expressed known genes, Con = control diet, SC06 = control diet containing 1 × 10^8^ CFU/g *Bacillus amyloliquefaciens* SC06, FC = fold change, n = 3 for each group.

**Supplementary Table 4:** Top 30 DEGs between the SC06+DQ and DQ groups

| **NCBI Gene ID** | **Gene name** | **up/down** | **Log_2_FC** | ***Q* value** |
| --- | --- | --- | --- | --- |
| 102159800 | *MVB12B* | up | 1.91 | <0.01 |
| 100525175 | *PLAC8* | up | 2.40 | <0.01 |
| 399538 | *PRDX6* | up | 1.64 | <0.01 |
| 100511886 | *FGFBP1* | up | 2.57 | <0.01 |
| 100154477 | *LGMN* | up | 1.57 | <0.01 |
| 100152549 | *PDZK1* | up | 1.37 | <0.01 |
| 100521318 | *GPT2* | up | 2.01 | <0.01 |
| 100524202 | *ZNF473* | down | -2.59 | <0.01 |
| 110261049 | *RDH13* | down | -2.34 | <0.01 |
| 100157523 | *GGT5* | down | -2.46 | <0.01 |
| 100519535 | *HNRNPH1* | down | -1.41 | <0.01 |
| 100153107 | *PAN2* | down | -1.90 | <0.01 |
| 100627929 | *FLYWCH2* | down | -2.92 | <0.01 |
| 492314 | *TAC3* | down | -2.17 | <0.01 |
| 100738561 | *ZNF251* | down | -2.25 | <0.01 |
| 100155485 | *OGT* | down | -1.69 | <0.01 |
| 100526033 | *FNBP4* | down | -2.03 | <0.01 |
| 100511789 | *SREK1* | down | -1.90 | <0.01 |
| 397569 | *CTSK* | down | -2.30 | <0.01 |
| 110261139 | *ZMYM6* | down | -1.90 | <0.01 |
| 100522160 | *HOXB3* | down | -3.10 | <0.01 |
| 100620926 | *TAF1* | down | -2.10 | <0.01 |
| 102164273 | *PIEZO1* | down | -2.63 | <0.01 |
| 100153622 | *SLC25A37* | down | -1.90 | <0.01 |
| 100511190 | *CCDC138* | down | -2.44 | <0.01 |
| 100517644 | *ZC3H7A* | down | -1.32 | <0.01 |
| 100153928 | *PRPF39* | down | -2.13 | <0.01 |
| 100158125 | *NAA16* | down | -1.76 | <0.01 |
| 100739606 | *EFCAB5* | down | -2.58 | <0.01 |
| 100628093 | *TOP1MT* | down | -3.32 | <0.01 |

DEGs = differentially expressed known genes, DQ = control diet plus diquat injection, SC06+DQ = control diet containing 1 × 10^8^ CFU/g *Bacillus amyloliquefaciens* SC06 plus diquat injection, FC = fold change, n = 3 for each group.

**Supplementary Table 5:** Significantly differential bacteria in the top 20 abundant bacteria between groups

| **Bacteria** | **Con (%)** | **DQ (%)** | **Log_2_FC** | ***P* value** |
| --- | --- | --- | --- | --- |
| p__Acidobacteria | 18.31 | 14.08 | -1 | <0.05 |
| f__Turicibacteraceae | 8.69 | 33.53 | 1.99 | <0.05 |
| g*__Clostridium* | 20.32 | 51.39 | 2.02 | <0.05 |
| g*__Turicibacter* | 8.69 | 33.53 | 1.99 | <0.05 |
|  | **Con (%)** | **SC06 (%)** |  |  |
| f__Ruminococcaceae | 47.01 | 8.67 | -2.44 | <0.05 |
| g*__Lactobacillus* | 6.51 | 29.89 | 2.18 | <0.05 |
|  | **DQ (%)** | **SC06+DQ (%)** |  |  |
| f__Pasteurellaceae | 6.90 | 22.44 | 1.68 | <0.05 |
| g*__Clostridium* | 51.39 | 26.29 | -2.50 | <0.01 |
| g*__Actinobacillus* | 3.70 | 18.63 | 2.33 | <0.05 |

Con = control diet, DQ = control diet plus diquat injection, SC06 = control diet containing 1 × 10^8^ CFU/g *Bacillus amyloliquefaciens* SC06, SC06+DQ = control diet containing 1 × 10^8^ CFU/g *Bacillus amyloliquefaciens* SC06 plus diquat injection, FC = fold change, n = 6 for each group.

**Supplementary Table 6:** Correlation coefficient (r) and *P*-value for correlation analysis between ER stress-related genes and bacterial genus abundance

| **Genes** | **Bacterial genus** | **r-value** | ***P*-value** |
| --- | --- | --- | --- |
| *SLC25A37* | *Clostridium* | 0.62 | <0.01 |
| *ERO1α* | *Clostridium* | 0.58 | <0.01 |
| *SOD3* | *Clostridium* | -0.52 | <0.01 |
| *eIF2α* | *Turicibacter* | 0.48 | <0.05 |
| *CHOP* | *Clostridium* | 0.48 | <0.05 |
| *PRDX6* | *Clostridium* | -0.48 | <0.05 |
| *ATF4* | *Clostridium* | 0.47 | <0.05 |
| *GGT5* | *Clostridium* | 0.47 | <0.05 |

SLC25A37 = solute carrier family 25 member 37, ERO1α = endoplasmic reticulum oxidoreductin 1α, SOD3 = superoxide dismutase 3, eIF2α = phosphorylation of eukaryotic initiation factor-2α, CHOP = C/EBP homologous protein, PRDX6 = peroxiredoxin 6, ATF4 = activating transcription factor 4, GGT5 = gamma-glutamyltransferase 5, n = 6 for each group.
